# Role of Growth Factors in Nasal Cartilage Development and Molding: A Comprehensive Review

**DOI:** 10.7759/cureus.67202

**Published:** 2024-08-19

**Authors:** Nikita Soni, Priyanka Niranjane, Akanksha Purohit

**Affiliations:** 1 Department of Orthodontics and Dentofacial Orthopaedics, Sharad Pawar Dental College and Hospital, Datta Meghe Institute of Higher Education and Research, Wardha, IND; 2 Neglected Tropical Diseases, Global Health Strategies, Delhi, IND

**Keywords:** nasal cartilage molding, cartilage growth and development, growth factors, cleft lip and palate (clp), chondrocytes

## Abstract

This review aims to investigate the properties of growth factors concerning the morphogenesis and development of nasal cartilage, which is fundamentally important for facial form and appearance. Since cartilage lacks a blood supply, it is more difficult to regenerate, as cartilage tissue obtains sustenance by diffusion. Cytokines are signalling molecules that control chondrocyte metabolism and extracellular matrix formation, which is required for cartilage development, homeostasis, and healing. Some craniofacial illnesses alter the composition of the cartilage and the structural organization of growth factors, allowing for moulding. TGF-β (transforming growth factor-β) encourages chondrocyte differentiation, whereas IGF-1 (insulin-like growth factor-1) stimulates cartilage-forming collagen synthesis and chondrocyte multiplication. We used the scoping review approach to present current research on the role of growth factors in the creation and architecture of nasal cartilage. We generally observed this structure before conducting specific experiments to determine the impact of growth agents on the development of chondrocytes and cartilage. Prominent findings increase our understanding of how growth factors influence the extracellular matrix, cell activities and features, and cartilage growth rate; all are critical for cartilage tissue development and repair. Research into growth factors and their physiological interactions with cartilage may help improve treatment's functional and aesthetic outcomes and our understanding of the origins and consequences of nasal congenital anomalies. This study emphasizes the importance of expanding knowledge and experience, as well as the use of growth factors in clinical practice, to stimulate cartilage development.

## Introduction and background

Cartilage is a framework of structures like the nose, ears, and joints. It does not have a direct blood supply as it is avascular, which makes damage repair more difficult. Unlike blood, cartilage gets nutrients from its peripheral tissues by diffusion [1]. Growth factors are complex hormones critical in coordinating cartilage health, growth, and repair, characterized by complex processes. The proteins acting as signal molecules are natural and secreted by cells and attach to specific receptors on the cells that are specifically involved, leading to a series of body defence reactions. The growth factors in cartilage mainly act as important regulators for the chondrocytes, which are the cells responsible for building and supporting the cartilage matrix web of fibres, allowing the structural support and resilience to be efficiently delivered [2].

Cartilage moulding is a method for gently repairing baby deformities, commandeering the particular flexibility of young cartilage, for instance, in the case of infants with cleft lip and palate. Nevertheless, it is not just a mechanical procedure over and above the limit of data processing. Growth factors form a crucial part of the moulding procedure for sustainability. Firstly, they increase the flexibility of cartilage. Thus, the specific growth factors represent the moulders of youth cartilage. Thus, they facilitate the control and reorganization of native tissue by modulating the constituents and organizing the extracellular matrix. Envision the extracellular matrix as a thick cable made out of filaments or fibres: growth factors determine the sort of those fibres and the configuration in which the fibres are presented, making the cartilage more flexible and more receptive to mould [3].

Secondly, growth factors enable cartilage growth to take place. In influencing the chondrocyte activity, they stimulate the synthesis of new cartilage matrix, facilitating the achievement of the intended shape. These factors directly lead to the stimulation of chondrocytes to make collagen, the major structural component of the matrix, and other necessities for forming more cartilaginous tissue. These growth-promoting factors help the moulded cartilage retain the new shape by filling the minor imperfections that would have been left in the preceding processes [4].

Thirdly, they contribute to cartilage tissue repair as precursors of growth factors in body tissues with restored functionality. These factors then equate to natural healers, which help in the migration and differentiation of cells at the site of injury, as well as the recruitment of cells to the patch and their subsequent functional chondrocytes. Such organization of the repair process contributes to the best result and the exclusion of complications that may occur due to minor injuries. It is possible to note several growth factors that are considered potential candidates for improving cartilage moulding. TGF-β (transforming growth factor-β) is involved in chondrocyte growth factor and differentiation and is responsible for a balanced number of cartilage-forming cells. IGF1 was understood to increase collagen output, a significant cartilage matrix structural protein. Researchers can achieve the maximum results using this technique by understanding the direct impact of these and other growth factors on nasal cartilage production and their interaction with moulding processes [3].

Growth factors are known for their importance, and their relevance is felt more when there is a modulated level of these factors. Other problems follow the regulation of growth factor signalling. Lack of sufficient growth factors inhibits flexibility and deforms the cartilage, making it more mould-resistant. It may be challenging to achieve a symmetrical pattern because the strategy requires more aggressive moulding, creating higher risks for the operation's complications. The difficulties arise from restricted cartilage development because of weak growth factor functionality. Sprouting the new cartilage tissue during moulding is essential in moulding the structure to the right shape and offering adequate support. The low activity of growth factors could be explained by the inability to form new cartilage and an asymmetrical appearance or structure instability in reshaping. Another factor that may be affected by low growth is delayed cartilage repair. In moulding, even minor injuries may cause potential complications such as infection or scar tissue formation if growth factors disrupt the organism's responses. This low level of growth factors finally affects the moulding procedure's form and functionality to an adverse effect [5].

The process of nasal cartilage moulding, described above, especially in cleft lip and palate patients, is quite different. This paper shows that the structure of the human nose and the possible features of its pathological growth require further consideration of the impact of growth factors. This paper is a review article that focuses on the complex connection between growth factors and chondrogenesis. Based on current knowledge, the future approach to applying growth factors to improve the cartilage moulding process is outlined to achieve better results in treating congenital nasal anomalies in infants, leading to improving their facial aesthetic and quality of life [6,7].

Methodology

Arksey and O'Malley (2005) and Levac et al. (2010) used the prescribed methodological procedure for a scoping review [8]. This scoping review sought to identify the state of knowledge on the mechanisms of growth factors in moulding nasal cartilage. The abovementioned approach offered a clear and systematic approach to the procedure, which aimed to find the existing literature and consolidate the existing knowledge. The primary research question for this scoping review was: How do growth factors influence the moulding of nasal cartilage?

Search Strategy

Given the objective of the present review, it remains essential to outline a basic common strategy of an electronic database search to identify relevant papers, which has no limitation on the year of publication and includes all the platforms like MEDLINE via PubMed, Google Scholar, and ScienceDirect. A combination of keywords was used to ensure a targeted approach: 'nasal cartilage', 'NAM', 'growth factors', 'cartilage', 'cleft lip and palate', and 'growth and development'.

An additional manual search was conducted through the reference lists of the chosen studies to supplement the computerized search and locate additional relevant publications that needed to be classified.

Study Selection

The retrieved articles were subjected to a rigorous selection process based on predetermined inclusion and exclusion criteria. Inclusion criteria were studies investigating the role of growth factors in cartilage growth and development, studies published in peer-reviewed journals, original research, in vivo and in vitro studies, literature reviews, and studies involving human, rodent, rabbit, and pig models, as these animals served as relevant models for cartilage development. Research on nasal chondrocytes using tissue engineering was also included. Exclusion criteria were studies not directly related to growth factors and nasal cartilage development, unpublished articles (e.g., theses, dissertations), surgical case studies, studies solely focused on nasal cartilage pathologies, and articles not published or translated into English.

Charting the Data

The study design and execution involved collaborative efforts among the three authors. The first author collected all relevant articles and drafted the manuscript. The second author modified and validated the content to ensure accuracy and coherence. The third author provided a final review by proofreading the manuscript, ensuring clarity and consistency. Relevant information from the included studies was gathered using a standardized data extraction form. This form captured vital details such as study design (randomized controlled trial, case-control study), methodology (in vivo, in vitro), research subjects (human infants, animal models), specific growth factors investigated, and outcomes related to cartilage moulding effectiveness. The extracted data were systematically charted and analyzed to identify recurring themes, patterns, and knowledge gaps. A narrative synthesis approach was employed to summarize and critically appraise the findings from the included studies, allowing for a comprehensive understanding of the current state of knowledge regarding the potential role of growth factors in enhancing the effectiveness of nasal cartilage moulding procedures.

PRISMA Flowchart

**Figure 1:**
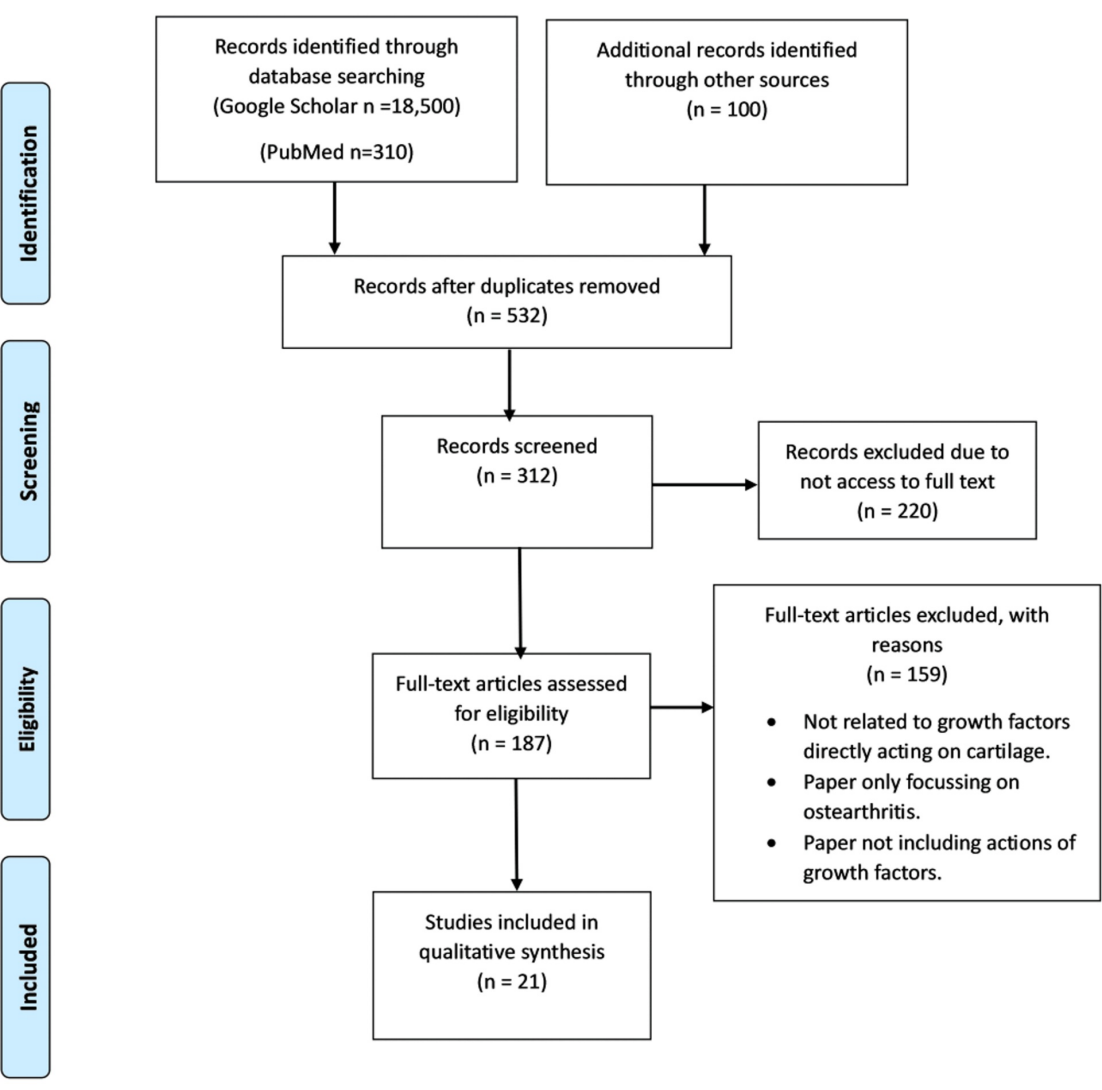
Preferred Reporting Items for Systematic Reviews and Meta-Analyses (PRISMA) flowchart showing the selection process for relevant studies.

The PRISMA flowchart depicted selecting relevant articles for the scoping review on the effects of growth factors on cartilage growth and development. PRISMA, which stands for Preferred Reporting Items for Systematic Reviews and Meta-Analyses, is a reporting guideline ensuring transparency and completeness in systematic reviews. The flowchart detailed the different stages involved in filtering the articles. Initially, researchers identified 18,810 articles (18,500 from Google Scholar and 310 from PubMed). After removing duplicates, the total was reduced to 532 articles. The eligibility of these articles was then assessed, resulting in the exclusion of 220 articles due to the unavailability of their full text. The full text of the remaining 312 articles was reviewed, and 159 articles were excluded for not being directly related to growth factors acting on cartilage, focussing only on osteoarthritis, or not including actions of growth factors. Ultimately, 21 articles were included in the qualitative synthesis, illustrating a transparent process for selecting articles for the scoping review (Figure 1).

## Review

Many discoveries of how growth factors affected cartilage growth and development were tracked in various successful articles shown in Table 1. Baddam et al. [8] performed a scoping review that included 86 articles selected from the database between 1963 and 2021, and it deals with the properties of nasal cartilage. Essentially, they noted that growth factors affected cartilage growth through factors that promoted cell division and deposition of matrix, an essential role in repair and regeneration. Thus, understanding the cross-talk between growth factors and extracellular matrix, such as collagen II and proteoglycans, was critical for cartilage homeostasis through the progression of cartilage development from birth to adulthood.

A paper by Gupta et al. [9] covered the genetics of nasal development in which growth factors, including sonic hedgehog (SHH), fibroblast growth factor (FGF), TGF-β, WNT, and bone morphogenetic protein (BMP), were described. It was established that these factors play a pivotal role in cell transformation, proliferation, differentiation, and chondrogenesis, shaping the nasal cartilage framework and guiding facial morphogenesis. Of all the genetic interactions, those between SOX9 and BMP4 were vital in chondrogenesis, especially for forming nasal cartilage and its ossification.

Wang et al. [10] conducted a study in 2015 looking at the TGF-β signalling network in cartilage formation and maintenance. They discovered that TGF-β was vital for chondrogenesis from mesenchymal condensation to chondrocyte terminal differentiation. It enhanced chondrocyte proliferation and sustained cartilage integrity by both Smad-dependent and Smad-independent pathways. Wenli Yu et al. [11] discussed cleft lip and palate genetics, stressing the significance of Fgf10 signalling through its receptor FgfR2b in epithelial proliferation during the early development of the palate. This signalling was essential in turning on the SHH expression in the epithelium, which excited the proliferation of the mesenchymal cells; such molecular interaction of Fgf and Tgf-β3 implicated in the palatal and nasal cartilage development.

The role of growth factors and cytokines in cartilage repair processes was examined by Gaissmaier et al. [12]. These factors, such as IGF-1, BMPs, PDGF, FGF-2, and VEGF, influence chondrocyte activity, differentiation, and matrix interactions; therefore, their roles were significant in understanding cartilage health and their possible uses in therapeutic applications. As with other cartilage types, these growth factors helped sustain the nasal cartilage shape, elasticity, and function via nutrition and cell signalling. In a study by Hall et al. [13], the authors described skeletal development, specifically TGF-β and BMPs, about cell condensation vital in cartilage formation. These growth factors affect cell adhesion, proliferation, and differentiation, including transcription factors such as Sox-9 expression, which influence condensation and chondrogenesis.

Finally, in their work on signalling pathways in cartilage repair, Mariani et al. [14] focused on FGF, TGF-β, BMP, and Wnt. Thus, they identified that FGFs stimulate hypertrophic differentiation and lead to cartilage breakdown, and BMPs and TGF-β stimulate anabolic processes and chondrocyte homeostasis. The TGF-β effect was mediated by inhibiting hypertrophy through activating the ALK5/Smad 2-3 signalling pathway, whereas BMP-2 enhanced both matrix production and hypertrophic differentiation, showing intricate interactions between cartilage biology and possible treatment approaches (Table 1).

**Table 1:** Relation of growth factors in cartilage growth.

S. No.	Author(s) and year	Aims/purpose	Study population and sample size	Methodology/methods	Intervention type/duration, comparator, outcome measures	Key findings that relate to the research question
1	Baddam et al. (2022) [8]	To review the properties of nasal cartilage from development to adulthood	Analyzed 86 articles from 1963 to 2021 on nasal cartilage properties	Scoping review using Arksey and O'Malley's framework	Growth factors significantly influence cartilage development, primarily by enhancing cell proliferation and matrix production, crucial for repair and regeneration.	Growth factors play a pivotal role in cartilage development by influencing growth rates and cellular characteristics, impacting ECM composition and maintaining cartilage integrity.
2	Gupta et al. (2020) [9]	To review the genetics of nasal development and morphological variation	nil	Articles were retrieved from the literature on the genetics of nose development and morphological variation, using keywords like 'craniofacial development', 'frontonasal development', 'nose and genetic variation', and selecting references from PubMed and Google Scholar.	Growth factors like SHH, FGF, TGFβ, WNT, and BMP direct maxillary pull, impacting facial growth and cartilaginous framework.	SHH, FGF, TGFβ, WNT, and BMP regulate nasal cell proliferation and differentiation, essential for chondrogenesis and nasal structure formation.
3	Wang et al. (2015) [10]	To study TGF-β signalling in cartilage development and maintenance	nil	A literature search identified relevant research on TGF-β signalling using academic databases like PubMed or Web of Science.	TGF-β influences chondrogenesis from condensation to differentiation, crucial for cartilage formation.	TGF-β pathways promote chondrocyte proliferation and maintain cartilage integrity, essential for skeletal development.
4	Wenli Yu et al (2009) [11]	To review the genetics and development of CLP	Reviews the causes and development of CLP, genetic and environmental influences	Various genetic studies, animal models, and developmental mechanisms	Fgf10 and Tgfβ3 impact epithelial proliferation and survival during palatal development.	Fgf10 and Tgfβ3 play crucial roles in epithelial survival, influencing mesenchymal cell fate and palatal development.
5	Gaissmaier et al. (2008) [12]	To study growth and differentiation factors for cartilage healing and repair	nil	nil	Growth factors (IGF-1, TGF-β, BMPs, VEGF, PDGF, FGFs) crucial for cartilage health.cartilage maintenance and repair.	Growth factors influence chondrocyte proliferation, differentiation, and metabolism, contributing to cartilage repair
6	Hall et al. (2000) [13]	To study condensations and initiation of skeletal development	Research done using mouse, chick, and murine embryos	Experiments in various organisms and cell cultures on skeletal development	TGF-β and BMPs regulate cell condensation and differentiation, crucial for skeletal development.	TGF-β and BMPs play pivotal roles in cartilage condensation and chondrogenesis, influencing nasal cartilage formation.
7	Mariani et al. (2014) [14]	To study signalling pathways in cartilage repair	Focus on molecular pathways in cartilage biology	Literature review, analysis of experimental data on molecular signalling pathways	FGFs, TGF-β, BMPs, Wnt regulate chondrocyte differentiation and cartilage homeostasis.	FGFs accelerate hypertrophic differentiation, while TGF-β and BMPs promote cartilage maintenance and matrix synthesis.

The various aspects of different growth factors being investigated in the works listed in Table 2 include their numerous effects on chondrocytes and cartilage growth, health, regeneration, and repair. Heine et al. [15] observed that TGF-β3 is highly abundant in the mouse embryo's cartilage and nasal tissue, demonstrating its importance in tissue development and cartilage formation. Kato et al. [16] proved that FGF positively influenced the colony's formation and proteoglycan synthesis in chondrocytes, confirming its role in preserving cartilage-specific traits. O'Keefe et al. [17] proved that TGF-β3 enhances DNA and collagen synthesis by chondrocytes, vital to cartilage health.

**Table 2:** Effects of growth factors on chondrocytes.

S. No.	Author(s) and year	Aims/purpose	Study population and sample size	Methodology/methods	Intervention type/duration, comparator, outcome measures	Key findings that relate to the research question
1	Heine et al. (1987) [15]	Investigate the role of TGF-β in mouse embryo development	Swiss-Webster mouse embryos	RNA extracted from mouse embryos and then analyzed for TGF-β1 presence.	Mouse embryo tissues derived from mesenchyme show high TGF-β1 levels throughout development. Immunohistochemical analysis shows TGF-β3 localization in connective tissues, including cartilage and nasal structures, during embryonic development.	TGF-β3 plays a crucial role in mesenchymal-epithelial interactions and tissue morphogenesis, particularly in cartilage formation and craniofacial development
2	Kato et al. (1987) [16]	Assess the effect of FGF on differentiated chondrocytes	Cell cultures	In vitro culture of differentiated chondrocytes	FGF significantly enhances colony formation in soft agar by chick embryo and rabbit chondrocytes, with colonies persisting for up to 40 days. Chondrocytes exposed to FGF maintain their differentiated state and synthesize cartilage-specific proteoglycans.	FGF promotes chondrocyte proliferation and maintains cartilage characteristics in vitro, highlighting its role in cartilage development.
3	O'Keefe et al. (1988) [17]	Analyze the effect of TGF-β on DNA synthesis by chick growth plate chondrocytes	Chick growth plate chondrocytes	Chondrocytes isolated and cultured to study DNA synthesis in response to TGF-β3	Exposure to TGF-β3 stimulates DNA synthesis and collagen synthesis in cartilage, influencing chondrocyte proliferation and matrix deposition.	TGF-β3 enhances DNA synthesis and collagen synthesis in chondrocytes, crucial for cartilage development and maintenance.
4	Hiraki et al. (1988) [18]	Assess the effect of TGF-β on rabbit growth plate chondrocytes	Young male New Zealand rabbits	Chondrocytes isolated from rabbit ribs and cultured to study DNA synthesis and glycosaminoglycan synthesis	Human platelet-derived TGF-β1 enhances DNA synthesis and glycosaminoglycan synthesis in rabbit growth plate chondrocytes.	TGF-β1 stimulates glycosaminoglycan synthesis and regulates DNA synthesis in rabbit growth plate chondrocytes, essential for endochondral bone formation.
5	Inoue et al. (1989) [19]	Investigate the effect of TGF-β on cartilage matrix proteoglycan synthesis	Rabbit rib chondrocytes	Isolated chondrocytes cultured in media with growth factors	TGF-beta enhances the synthesis of cartilage-characteristic proteoglycan in chondrocyte cultures, particularly in the presence of FGF.	TGF-beta promotes cartilage-specific proteoglycan synthesis, crucial for maintaining cartilage matrix integrity.
6	Rosa et al. (2014) [20]	Enhance the structure and properties of scaffold-free engineered auricular cartilage constructs using growth factor stimulation	Auricular chondrocytes from rabbits	Cultured in a bioreactor with growth factors (IGF-1, insulin, TGF-b1)	Insulin and IGF-1 enhance growth and properties of auricular cartilage constructs, mimicking native tissue characteristics.	Growth factors like IGF-1 and Insulin improve cartilage tissue thickness, mechanical properties, and biochemical composition in engineered constructs.
7	Hallett et al. (2019) [21]	Explain growth plate chondrocytes: Skeletal Development, Growth and Beyond	Postnatal growth plates in rabbits and mice	Lineage tracing, ablation, and cell transplantation methods	Various growth factors regulate the proliferation and differentiation of growth plate chondrocytes, influencing longitudinal bone growth.	Growth factors including BMP, FGF, TGF, EDGF, IHH play crucial roles in growth plate chondrocyte function and skeletal development.
8	Khan et al. (2018) [22]	Investigate how fibroblast growth factor 2 promotes regeneration of cartilage by attracting mesenchymal stem cells to the site of cartilage injury	10-week-old male DBA1 wild type (WT) or FGF2-/- mice	In this study, researchers investigated the role of FGF2 in mice with focal cartilage defects, analyzing mesenchymal stem cell migration, adhesion, and chondrogenesis.	Intervention type/duration: FGF2 knockout in DBA1 mice. Comparator: Wild-type DBA1 mice. Outcome measures: cartilage repair score, MSC migration and adhesion, in vitro chondrogenesis.	FGF-2 helps repair cartilage by attracting mesenchymal stem cells to the injury site.

The work by Hiraki et al. [18] indicated that TGF-β played multifunctions in cartilage biology; it stimulated glycosaminoglycan synthesis and regulated DNA synthesis in growth-plate chondrocytes, according to the study conducted. Thus, several previous studies, including Inoue et al. [19], pointed out that TGF-β improved chondrocytes by proteoglycan synthesis, which is vital for the cartilage matrix.

The study by Rosa et al. [20] identified that IGF-1 enhanced the quality of scaffold-free engineered cartilage constructs, whereas TGF-β1 reduced the quality. Hallett et al. [21] stated that growth factors like VEGF were central to endochondral ossification and cartilage-to-bone conversion. Lastly, Khan et al. [22] observed that FGF2 was also involved in cartilage repair since it recruited mesenchymal stem cells to the injury sites while having a reparative role. These studies provided a detailed picture of the functional importance of growth factors in chondrocyte biology and cartilage formation (Table 2).

In Table 3, Copray et al. [23] designed the purpose to investigate the aspect of nasal septal cartilage regarding the ability of its growth in a cell culture. This cross-sectional study harvested 66 four-day-old Wistar rats' nasal septal cartilage and cultured them in vitro for 10 days. The intervention referred to culturing rat nasal septal cartilage explants, which were four days old, in a serum-free medium for ten days. The approach used to establish the research findings was quantitative, and the measures used included cartilage growth rate, cell proliferation, and matrix synthesis. Several important observations were made: the nasal septal cartilage demonstrated significant volume increase, length, and thickness and maintained appropriate shape. Rates of cell proliferation were reduced over time. They were maximal at the septo-ethmoidal junction, while matrix synthesis was most significant at the junction and midline septum, as observed in vivo.

Iwata et al. [24] studied the mechanism of TGF-β signalling for palate development in the case of CL/P in humans. Culture samples included TGF-beta, bFGF, BMP-2, and IGF-1 for seven days. The study demonstrated that TGF-β is involved in the chondrogenic differentiation of MSCs, detailing cell fate determination, propagation, and differentiation via SMAD-dependent and independent pathways. Oral-nasal axes governed the cartilage formation based on the difference in the gene expressions that regulate the role of the formation of nasal cartilage compared to other cartilages.

Huber et al. [25] researched reconstructing the nasal septal defects in rabbits, and the study involved 24 New Zealand white rabbits aged 14-16 weeks. Decellularized porcine nasal cartilage scaffolds (DNSCs) were used in the study, and it exposed the PDGF-BB to investigate the response in terms of uptake and release. For seven days, TGF-beta, bFGF, BMP-2, and IGF-1 stimulated chondrocyte culture. Scaffolds with PDGF-BB showed decent chemotactic activity and stable cytokine production capabilities, stimulating perichondrium and adjacent cartilage into neocartilage formation. This approach also helped to maintain the structural integrity. It averted considerable shrinkage in the scaffold and was supplemented with moderate inflammation, suggesting clinical applicability for nasal septum reconstruction.

The effect of growth factors on human nasal septal chondrocytes was considered by Richmon et al. [26] from a sample of six patients, of whom four were male and the remaining two female. The experiment was conducted weekly with chondrocytes cultured at different concentrations using growth factors like TGF-beta and FGF-2. The combination of TGF and FGF was more effective in influencing matrix production and cell proliferation. As a result, there was a clear and significant interaction between the growth factors. Using serum-free conditions ensured control, high cohesiveness, and repeatability of the results and might have fewer immunologic threats than using the serum-containing media.

Bujía et al. [27] investigated growth factors that regulate the actions of chondrocytes in the human nasal septum. TGF-beta and bFGF were introduced to the cell culture, and the groups that were not stimulated were used as controls. Protein and proteoglycan synthesis, as well as type II collagen, were measured. These outcomes proved that the cell proliferation rate was most stimulated by bFGF, with the stimulation caused by TGF-beta being followed and EGF being the least. These growth factors acted synergistically, and using them simultaneously encouraged a six-fold increase in cell numbers, morphogenesis, and chondrocytes that can be harnessed for efficient cartilage propagation and possible application in reconstructive surgery.

Alexander et al. [28] described the use of IGF-1 and GDF-5 in enhancing the development of tissue engineering human nasal septal cartilage. It was a study in which sampling was done via isolating chondrocytes from human septa and culturing them in alginate beads with various growth factors. The intervention consisted of a two-week alginate beads culture and a four-week ARC construct culture with/without IGF-1 and GDF-5. Some of the findings elaborated that these growth factors promoted chondrogenesis, boosted the deposition of glycosaminoglycan and type II collagen in the generated constructs, and improved the mechanical properties of neocartilage tissue. This approach outlines a clear way forward for clinical applications in tissue engineering that is potentially safer and more effective than foetal bovine serum (Table 3).

**Table 3:** Growth factors' role in nasal cartilage.

S. No.	Author(s) and year	Aims/purpose	Study population and sample size	Methodology/methods	Intervention type/duration, comparator, outcome measures	Key findings that relate to the research question
1	Copray et al. (1986) [23]	Analyze the growth potential of nasal septal cartilage in vitro	Nasal septal cartilage from 66 four-day-old Wistar rats	Nasal septal cartilages from young rats were cultured in vitro for up to 10 days to study their growth and metabolism.	Intervention: Four-day-old rat nasal septal cartilage explants cultured in serum-free medium for 10 days (vs. in vivo controls). Comparator: None (in vitro vs. natural growth). Outcome measures: Cartilage growth rate, cell proliferation, and matrix synthesis.	In vitro, nasal septal cartilage acts as struts akin to long bone epiphyseal cartilage, suggesting its role in midfacial growth involves both responding to and potentially influencing osseous separation. Nasal septal cartilage in vitro shows significant volume growth, primarily lengthwise, maintaining shape. Proliferative activity decreases over time, highest at the septo-ethmoidal junction. Matrix synthesis peaks at the septo-ethmoidal junction and central septum, mirroring in vivo patterns.
2	Iwata et al. (2011) [24]	Mechanism of TGF-β signaling during palate development	Humans with cleft lip with or without cleft palate (CL/P)	Cells were cultured in various combinations of TGF-beta, bFGF, BMP-2, and IGF-1 for seven days.	Intervention: Various combinations of TGF-beta, bFGF, BMP-2, and IGF-1 for seven days. Comparator: Unstimulated cells in serum-free media or 2% FBS were used as controls. Outcome measures: Cell proliferation and GAG content.	TGF-β signaling initiates SMAD-dependent and SMAD-independent pathways, crucial for cell fate determination and differentiation in cartilage development. Differential gene expression along oral-nasal axes controls cartilage development, highlighting regulatory mechanisms for nasal cartilage distinct from other cartilaginous structures.
3	Huber et al. (2022) [25]	Regenerate nasal septal defects in rabbits	24 New Zealand white rabbits	Decellularized porcine nasal cartilage scaffolds (DNSC) were prepared and tested for PDGF-BB uptake and release, and biocompatibility in rabbits.	Intervention: Chondrocytes cultured with PDGF-BB-loaded DNSCs. Comparator: Unstimulated cells in serum-free media or 2% FBS were used as controls. Outcome measures: Cell proliferation and GAG content.	PDGF-BB-loaded scaffolds demonstrated effective chemotactic activity and stable cytokine release, promoting neocartilage formation from perichondrium and neighboring cartilage. This approach supports in situ tissue engineering, avoiding donor site morbidity associated with cell-based therapies, and suggests potential for clinical applications in nasal septum reconstruction.
4	Richmon et al. (2005) [26]	Effect of growth factors on cell proliferation, matrix deposition, and morphology of human nasal septal chondrocytes	Human nasal septal chondrocytes from a sample of six patients (4 male, 2 female)	Isolated nasal septal chondrocytes were cultured with growth factors to assess proliferation and GAG content.	Intervention: Chondrocytes cultured with various growth factors for 7 days. Comparator: Unstimulated cells in serum-free media or 2% FBS were used as controls. Outcome measures: Cell proliferation and GAG content.	The study explores tissue engineering of cartilage, focusing on growth factors like TGF-1 and FGF-2 to enhance chondrocyte proliferation and matrix synthesis. Tissue engineering involves isolating chondrocytes from nasal septum or auricle, proliferating them with growth factors like TGF-ß and FGF-2, which enhance matrix synthesis and maintain chondrocytic morphology. Low-dose fetal bovine serum promotes cell proliferation effectively, yet serum-free conditions offer greater control and reproducibility, highlighting the importance of growth factor interactions. Combinations of TGF-1 and FGF-2 synergistically enhance cell proliferation, suggesting potential for serum reduction in culture media for optimized cartilage tissue engineering.
5	Bujía et al. (2009) [27]	Effect of growth factors on cell proliferation by human nasal septal chondrocytes cultured in monolayer	Chondrocytes from nasal septal cartilage	Chondrocytes were cultured with TGF-beta and bFGF to measure protein and proteoglycan synthesis.	Intervention: TGF-beta and bFGF were added to chondrocyte cultures. Comparator: Unstimulated cells were used as control. Outcome measures: Protein and proteoglycan synthesis.	Growth factors like TGF-beta, bFGF, and EGF significantly enhance chondrocyte proliferation in vitro, crucial for cartilage engineering. bFGF showed the most potent effect, followed by TGF-beta, while EGF had minimal impact. Combining these factors resulted in synergistic effects, promoting up to sixfold increases in cell proliferation, thus facilitating efficient cartilage amplification for potential transplant applications in reconstructive surgery.
6	Alexander et al. (2010) [28]	Insulin-like growth factor-I and growth differentiation factor-5 promote the formation of tissue-engineered human nasal septal cartilage	Human nasal septal cartilage donors	Chondrocytes were cultured in alginate beads with IGF-1 and GDF-5 to assess cartilage formation.	Intervention: Alginate beads with IGF-1 and GDF-5 for four weeks. Comparator: Different combinations of growth factors and serum conditions (2% vs 10% HS). Outcome measures: Cell-associated GAG accumulation, construct weight, thickness, DNA content, collagen content (Type I & II), and a compressive modulus.	The study demonstrates that growth factors like IGF-1 and GDF-5, when added to culture medium supplemented with human serum, significantly enhance chondrogenesis of human nasal septal chondrocytes. This results in increased accumulation of glycosaminoglycans (GAG) and type II collagen, improving mechanical properties in engineered neocartilage constructs, thus potentially offering a promising strategy for clinical applications in tissue engineering.

Discussion

The development and growth of cartilage involve a complex process through the interaction of a series of growth factors responsible for the harmony of the cellular activities that are important in the stability of the cartilage tissues and their functions. However, TGF-β played a varied role among the growth factors in chondrogenesis, the development of cartilage from mesenchymal stem cells [29]. TGF-β acts through signal transduction pathways via Smad-dependent and Smad-independent pathways. In chondrogenic differentiation, TGF-β stimulated the ovaries of cartilage-specific large molecules like collagen and glycosaminoglycans, which are the building blocks of cartilage [30]. It was also involved in the processes of cell division and the balance of the matrix's synthesis and degradation, which were essential for chondrocyte maintenance.

Research has established that TGF-β acts as a dose-dependent function and influences DNA synthesis and cell growth depending on the other growth factors, including FGF, IGF, and PDGF [31]. Furthermore, combining TGF-β with FGF and other growth factors favoured chondrogenic differentiation and matrix deposition. This interaction brought out a complex web of cartilage growth and development, a function seen to be played by TGF-β [32]. It is therefore suggested that TGF-β signal dysregulation could result in developmental disorders or disorders affecting cartilage, such as osteoarthritis.

TGF-β, BMP, FGF, and other factors influence the shape of nasal cartilage during its development since the growth factors drive mesenchymal condensation during cartilage formation and chondrogenic differentiation. For example, BMP signalling prompted both the processes of chondrogenesis and osteogenesis, which are required for the framework formation of nasal bone and cartilage [33]. Various experiments have proved the implications of transcriptional factors such as SOX9 in controlling collagen and aggrecan, which are crucial for nasal cartilage formation [34]. Any disturbances of these developmental processes, for instance, through ALX1, ALX3, ALX4, or SOX9 gene mutations, can result in craniofacial disorders like frontonasal dysplasia, cleft palate, or other abnormalities of the nasal contour, emphasizing the highly regulative nature of developmental morphogenesis of nasal structure [35].

Understanding the fundamental biological mechanisms of the signalling pathways and molecular mechanisms that drive cartilage production and growth is crucial for healthcare providers. The signalling pathway information may help treat cartilage defects and degenerative diseases and may use growth factors to promote the repair and regeneration of cartilage. Ultimately, TGF-β plays a crucial role in cartilage growth and development by regulating matrix formation, chondrogenic differentiation, and homeostasis. Their interactions with other signalling molecules and genes were critical for bone and cranial morphogenesis and differentiation, demonstrating the delicate balance required for cartilage growth and function [4].

This review comprehensively examines growth factors in cartilage moulding, particularly for nasal cartilage in cases of congenital anomalies like cleft lip and palate. Its strengths lie in its systematic approach to synthesizing diverse studies, which offers a broad understanding of how various growth factors influence cartilage development and repair. Including multiple growth factors and their mechanisms, alongside a detailed methodology including PRISMA flowchart transparency, strengthens the review's reliability. However, the review's limitations include the variability in study designs and methodologies of the included articles, which may affect the consistency and comparability of findings. In addition, the review's focus on specific congenital conditions may need to fully address the broader implications of growth factors in other cartilage disorders or developmental stages.

## Conclusions

Various growth factors are interdependent, including TGF-β, BMPs, FGFs, and IGFs, which actively regulate or modulate various cartilage growth and development types by different signalling pathways. It can be concluded that TGF-β acts as a significant component in the process of chondrogenesis in regulating the production of cartilage-specific macromolecules, controlling cell division, and mediating the homeostasis of the extracellular matrix. These signalling pathways were Smad-dependent and Smad-independent and worked in harmony with FGFs and IGFs to promote chondrocyte differentiation and matrix formation. BMPs promoted chondrogenesis and osteogenesis, which are critical for skeletal development, while FGFs surged hypertrophic differentiation and cartilage repair through the recruitment of MSCs (mesenchymal stem cells). IGFs maintain cartilage organization and its mechanical characteristics by promoting GAGs and collagen synthesis. Altogether, growth factors regulate the cellular processes essential for cartilage formation, maintenance, and repair, and they might be helpful for cartilage pathology therapy and tissue engineering. These pathways were indeed significant for the understanding of fundamental biological processes and establishing the notion of the proper regulation to support cartilage formation and function.
